# Protective effect of curcumin on TNBS-induced intestinal inflammation is mediated through the JAK/STAT pathway

**DOI:** 10.1186/s12906-016-1273-z

**Published:** 2016-08-20

**Authors:** Xingxing Zhang, Jian Wu, Bo Ye, Qiong Wang, Xiaodong Xie, Hong Shen

**Affiliations:** 1 Department of gastroenterology, the affiliated hospital of Nanjing university of Chinese medicine, Jiangsu province hospital of traditional Chinese medicine, No 155 Hanzhong Road, Nanjing, 210029 China; 2 Central Laboratory, the affiliated hospital of Nanjing university of Chinese medicine, Jiangsu province hospital of traditional Chinese medicine, No 155 Hanzhong Road, Nanjing, 210029 China; 3 Pharmacology laboratory, the affiliated hospital of Nanjing university of Chinese medicine, Jiangsu province hospital of traditional Chinese medicine, No 155 Hanzhong Road, Nanjing, 210029 China; 4 Department of surgery, the affiliated hospital of Nanjing university of Chinese medicine, Jiangsu province hospital of traditional Chinese medicine, No 155 Hanzhong Road, Nanjing, 210029 China

**Keywords:** Curcumin, Intestinal inflammation, M1/M2, JAK/STAT pathways, Apoptosis

## Abstract

**Background:**

Curcumin displays a protective role in rat models of intestinal inflammation. However, the mechanism of how curcumin affects on intestinal inflammation is less known. The purpose of the current study is to explore the signal pathway in which the curcumin protecting rat from intestinal inflammation.

**Methods:**

The intestinal inflammation rat models were made by TNBS treatment. Curcumin was added to their diet 5 days before the TNBS instillation. After that, body weight change, score of macroscopic assessment of disease activity and microscopic scoring were utilized to analyse the severity of the induced inflammation. In addition, the level of pro-inflammatory cytokines and anti-inflammatory were detected to determine the effect of curcumin on intestinal inflammation. The JAK/STAT pathway of pro-inflammation response was also evaluated. Finally, the impact of curcumin on apoptosis in intestinal inflammation was assessed by TUNEL staining.

**Results:**

Rats pretreated with curcumin significantly reversed the decrease of body weight and increase of colon weight derived from TNBS-induced colitis. Histological improvement was observed in response to curcumin. In addition, curcumin attenuated TNBS-induced secretion of pro-inflammatory cytokines and M1/M2 ratio, while stimulated the secretion of anti-inflammatory cytokines. The inhibition of pro-inflammation response was mediated by SOCS-1, which could efficiently suppress JAK/STAT pathways. Furthermore, curcumin efficiently suppressed the TNBS-induced apoptosis, and reduced the accumulation of cytochrome C in cytosol.

**Conclusion:**

The anti-inflammatory effect of curcumin is realized by enhancing SOCS-1 expression and inhibiting JAK/STAT pathways. Curcumin also plays an anti-apoptotic role in TNBS-induced intestinal inflammation. We propose that curcumin may have therapeutic implications for human intestinal inflammation.

## Background

Inflammatory bowel diseases (IBD), including ulcerative colitis and Crohn’s disease, are caused by combined the effect of associated genes with complicated environmental factors [1]. From the point of biological processes, IBD susceptibility is derived from various physiological changes, such as dysregulated immunoregulation, mucosal barrier integrity and microbial recognition, clearance and/or homeostasis [2]. No cure can be used for IBD at present, however, the inflammatory process involved in IBD can be attenuated through some therapies, so as to improve the signs and symptoms in IBD and induce long-term remission hopefully.

Turmeric (also called *Curcuma longa*) is an Indian spice and extracted from the rhizomes of the plant. For a long history, turmeric has used as Ayurvedic medicine and a treatment for inflammatory conditions [3]. The main ingredient in turmeric is curcumin, also called diferuloymethane. Previous studied have demonstrate that curcumin, as highly pleiotropic molecule can interact with an abundance of molecular targets involved in inflammation regulation [3].

The mechanism of curcumin regulating the inflammatory response is complex. Studies have shown that with curcumin treatment, the activities of cyclooxygenase-2 (COX-2), lipoxygenase, and inducible nitric oxide synthase (iNOS) enzymes decrease, the protein kinase (MAPK) signaling is inhibited and the levels of the inflammatory cytokines, including tumor necrosis factor-alpha (TNF-α), interleukin (IL) −1, −2, −6, −8, and −12, monocyte chemoattractant protein (MCP), and migration inhibitory protein are downregulated [3, 4]. It is well known that global transcription factor NF-kB is response to various signaling pathways, especially the regulation of inflammation. Curcumin is thought to down-regulate COX-2 and iNOS expression mediated by NF-kB, thus improving the inflammatory process and tumorigenesis [5, 6].

Originally, Janus kinase (JAK)/signal transducers and activators of transcription (STAT) signaling is identified as the signaling pathway for interferons (IFNs). Afterwards, it is identified to make contributions to inflammation mediated by the immune responses of a dozen of cytokines, the actions of various growth factors and hormones [7]. As suppressor of cytokine signaling, SOCS-1, is an important negative regulators of JAK/STAT signaling shown in myeloproliferative neoplasms (MPNs) and leukemia [8]. The induction of SOCS-1 leads to sharply downregulate JAK2/STAT signaling and the mRNA levels of target genes which are directly regulated by JAK2 [9, 10]. Previous work has demonstrated the inhibitory action of curcumin on JAK-STAT signaling contributes to its anti-inflammatory activity, and curcumin can markedly inhibit the phosphorylation of STAT1 in brain microglia [11]. Furthermore, curcumin can also increase the expression of SOCS-1 through inhibiting class I histone deacetylases in myeloproliferative neoplasms [12]. However, whether the effect of curcumin on anti-inflammatory activities in TNBS-induced intestinal inflammation is mediated by STAT1 and SOCS-1 is less known.

In this work, the results of our study demonstrated that curcumin had anti-inflammatory and anti-oxidative effects in TNBS-induced intestinal inflammation rat models, which inhibited the inflammation in colon, and caused an attenuated M1/M2 ratio. We also clarified that curcumin was involved in intestinal inflammation through elevating the expression of SOCS-1 and suppressing the phosphorylation of STAT1. Furthermore, curcumin reduced TNBS-induced apoptosis was through the mitochondrial pathway and declined the accumulation of cytochrome C in cytosol.

## Methods

### Animal studies

Sprague Dawley male rats (180–200 g) were maintained in accordance with the guidelines of the Committee for Animal Research of Nanjing University of Chinese Medicine. This experiment was manipulated in line with the Regulations for the Administration of Affairs Concerning Experimental Animals. All animal experiments were approved by Animal Ethics Committee in Nanjing university of Chinese medicine. A total number of 78 rats were used. For body weight and colon tissue weight measurement, three groups (control group, TNBS group, and curcumin + TNBS group) of 18 rats in total were used. 60 rats were randomly allocated into ten groups. Group 1: TNBS group, samples collected 6 h after treatment; Group 2: TNBS group, samples collected 12 h after treatment; Group 3: curcumin + TNBS group, samples collected 6 h after treatment; Group 4: curcumin + TNBS group, samples collected 12 h after treatment; Group 5: control group to Group 1 ~ 4; Group 6: TNBS group, samples collected 1 day after treatment; Group 7: TNBS group, samples collected 2 day after treatment; Group 8: curcumin + TNBS group, samples collected 1 day after treatment; Group 9: curcumin + TNBS group, samples collected 2 day after treatment; Group 10: control group to Group 6 ~ 9.

To estabilish the colitis rats model, 20 mg/ml 2, 4, 6-trinitrobenzene sulfonic acid (TNBS) (Sigma-Aldrich, St. Louis, MO, USA) was dissolved in 50 % ethano. A rubber infusion tube was positioned 8 cm from the anus to instill TNBS into the lumen of the colon. Keep the rats vertically for at least 1 min to ensure the TNBS enter entire colon. As control, the normal group also were delivered 50 % ethanol with an equal volume. One of the rats treated with TNBS were selected and sacrificed after 4 days treatment to evaluate the colitis. Curcumin (purity, 95 %) was purchased from BDH Company in England. For curcumin treatment groups, a concentration of 0.25 % curcumin was added to their diet at 5 days before the TNBS instillation. After death of the animals, the inflamed mucosa were harvest to do macroscopic damage scores, hematoxylin and eosin (H&E) staining, Western Blot, and qRT-PCR analysis [13, 14].

### Parameters utilized to analyse the severity of the induced inflammation

Body weight change: the weight of each animal was measured, starting on the day before TNBS treatment, and the change in weight was calculated as a percentage, the baseline (marked as 100 %) being taken as the weight on the day of the TNBS challenge.

The method of macroscopic damage scores was described previously [15]. Briefly, 1: no damage; 2: focal hyperemia no ulcers; 3: ulceration without hyperemia or bowel wall thickening; 4: ulceration with inflammation at one site, but not more than two sites of ulceration and inflammation and not more than two major sites of ulceration and inflammation, or one site of ulceration extending >1 cm along length of colon; 5: if damage covered >2 cm along length of colon.

The standard of microscopic scoring followed previous method [16]. Colons were isolated and fixed with 4 % formalin. After embedded in paraffin, 4 μm thick sections were obtained. Following the H&E staining, microscopic scoring was performed by two different pathologists blinded to evaluate. This score grades the severity of the lesion from 0 to 6 on the basis of the extent of inflammatory infiltrate (superficial mucosal to deeper lesions into the muscularis propria), extent of ulceration, and the presence or absence of necrosis. Neutrophils were numbered in more than eight highpower fields in six rats in groups.

### Immunohistochemistry

Before experiment, xylene was used to dewax paraffin-embedded colonic tissue every 5 min for twice. Afterwards, the tissue was rehydrated in gradient concentration of ethanol (100–70 %) every 5 min for three times. And then it was rehydrated in PBS for 30 min. The staining kit (Dako Real, Cat. K5001, Dako Cytomation) was used according to manufacturer’s instructions.

The primary antibodies were used as following: anti-CD11c (ab52632, clone EP1347y, Abcam, Cambridge, UK), anti-CD163 (MAB1652, clone K20-T, Abnova, Taipei City, Taiwan). The biotinylated immunoglobulins were used as secondary antibody and stained with 3,3’-diaminobenzidine (DAB) (Dako Cytomation) substrate solution. The hematoxylin (Dako Cytomation) was used to counterstain the nucleus.

### Myeloperoxidase activity assay

A myeloperoxidase assay kit (HK210; Hycult biotechnology, Netherlands) was used to measure myeloperoxidase activity. Colon tissues were thoroughly washed with cold PBS to remove faeces and weighed in an Eppendorf tube. The colon lysis buffer containing 200 mM NaCl, 5 mM EDTA, 10 mM Tris, 10 % glycerol, 1 mM PMSF, 1 μg/ml leupeptide, 28 μg/ml aprotinine, pH7.4 was prepared and kept cool on the ice before use. Every 10 mg of tissue added 200 μl colon lysis buffer, and homogenized in tissue homogenizer at 4 °C. The supernatant was isolated by centrifuging and detected by using a microplate reader (Biotek) in 450 nm.

### Western blot

The homogenization buffer contained 20 mM MOPS, 50 mM β-glycerophosphate, 5 mM EDTA, 1 mM DTT, 1 mM sodium vanadate, 1 mM PMSF, and 50 mM NaF. Tissue was mixed in the homogenization buffer and sonicated on the ice. The protein concentration in the supernatent isolated by centrifuging was determined by the Bradford assay (Thermo). Proteins were separated with SDS-PAGE and then transferred to PVDF membranes on the ice (Bio-Rad). The membranes was incubated with appropriate primary antibody overnight at 4 °C. After three times washed, the appropriate secondary antibody was added and incubated for 1 h at room temperature. After three times further washing, the enhanced chemiluminescence detection buffer (Amersham) was dripped on the membrane. Signal was detected by Chemiluminescence Apparatus (Bio-Rad, CA). GAPDH was used as reference. All experiments were repeated at least three times. The following antibodies were used: SOCS-1, STAT1 and p-STAT1 (Cell Signaling Technology, Beverly, MA).

### Isolation of RNA and qRT-PCR

The colon grinded with liquid nitrogen and RNA was isolated with TRIzol (Life Technologies). The quality of the RNA was determined on a 1 % agarose gel for 2 h at 60 V. Reverse transcription (RT) was operated with the Super Script III First-Stand Synthesis System (Invitrogen). To detect the mRNA level of the target genes, quantitative real-time PCR (qRT-PCR) was performed using a SYBR Premix Ex TaqII (TaKaRa). The qRT-PCR procedure was according to the manufacturer’s instructions from TaKaRa and ran with ABI PRISM7500 Fast Real-Time PCR System. The primers of iNOS, Fizz-1, STAT1, SOCS-1 and β-actin (control) were listed in Table 1.

**Table 1:** Rat primers for real-time PCR

Gene	Forward Primer	Reverse Primer
iNOS	GATTTTTCACGACACCCT	GGTCCTCTGGTCAAACTC
Fizz-1	CAACAGGATGAAGACTGCAACCT	GGGACCATCAGCTAAAGAAG
STAT1	AGGTCCGTCAGCAGCTTAAA	CGATCGGATAACAACTGCTT
SOCS-1	CCGTGGGTCGCGAGAAC	AAGGAACTCAGGTAGTCACGGAGTA
β-actin	TTCAACACCCCAGCCATGT	CAGTGGTACGACCAGAGGCATA

### ELISA for cytokines

Supernatants of biopsy cultures were used to ELISA detection after a transient centrifugation. The cytokines including TNF-α, IFN-γ, TGF-β, IL-13 (R&D Systems, Abingdon, UK) and IL-10 (Immunotools, Friesoythe, Germany) were selected. Each sample was tested in duplicate against the appropriate standard and optical densities measured by microplate reader (BioRad, Hemel Hempstead, UK).

### Cytochrome C releasing assay

To detect mitochondria cytochrome C release, tissue was homogenized, and supernatant cytosol fraction separated by centrifugation. The detection method was following the manufacturer’s instructions (Abcam, AB65311).

### TUNEL assay

TUNEL staining was performed with an Apop Tag® Fluorescein in situ Apoptosis Detection kit (millipore) according to the manufacturer’s instructions. Briefly, the tissue cryosections were fixed with 1 % paraformaldehyde for 10 min at room temperature. Afterwards, the sections were thoroughly rinsed with PBS. The terminal deoxynucleotidyl transferase enzyme mixture was used to treat sections at 37 °C for 1 h, and kept them in a humidified chamber without light. Next, the sections were incubated with the anti-digoxigenin antibody conjugated to fluorescein for 30 min without light. At last, sections were viewed with laser confocal microscopy.

To identify the necrosis, the method was described previously [17]. Briefly, a concentration of 0.1 μg/ml PI were used to treat the 10 μm sections for 10 min. The nuclei was counterstained with DAPI.

## Results

### Curcumin improves macroscopic tissue damage in TNBS-induced IBD

**Fig. 1:**
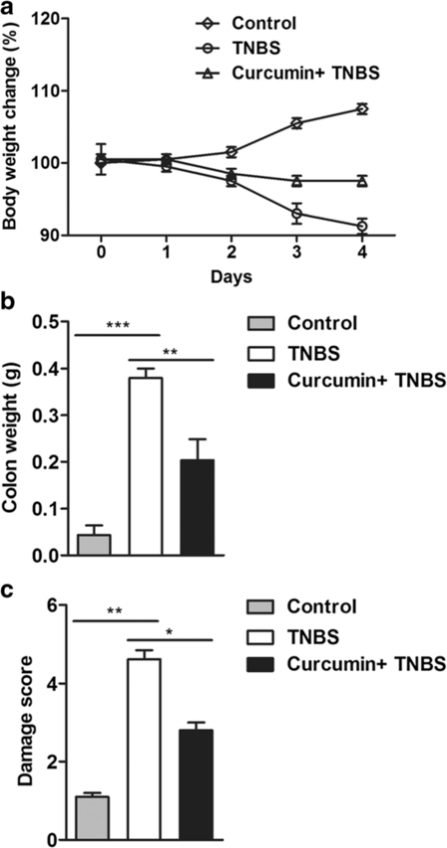
Curcumin protects colonic tissue from TNBS-induced damage. **a** The weight of rats were recorded daily after TNBS treatment. The data shown were representative. **b** The colons were weighed after 5-day TNBS challenge. Pretreatment with curcumin significantly decreases the weight of colons. **c** After curcumin pretreatment, the macroscopic damage was improved. *P < 0.05, **P < 0.01, ***P < 0.001

Body weight is an important marker for IBD in rats. The body weight of DNB (dinitrobenzene sulfonic acid) - induced IBD rats were lower than normal rats [13]. The normal rats had a normal diet and their body weight increased during experiments. However, TNBS caused stagnated body weight of rats in contrast with the weight gain in control group (Fig. 1a). Moreover, TNBS treatment group showed significant phenotypes of colitis in rats, including bloody diarrhea, wasting conditions, and dull reaction. We further measured the weight of colon tissue after 5-day TNBS challenge which located 5 cm proximal to the rectum. The weight of colon tissue gained with TNBS treatment (Fig. 1b). However, rats pretreated with curcumin significantly improved the decrease of body weight and gain of colon weight associated with TNBS-induced colitis (Fig. 1a and b). The increase weight of colon was a consequence of apparent mucosal thickening [18], therefore, curcumin can efficiently decrease the thickening derived from inflammation.

Next, macroscopic score was performed to assess the damage degree. As indicated in Fig. 1c, an increased inflammation in response to TNBS-induced colitis for 5 days. In contrast, attenuated inflammation with curcumin pretreatment. Thus, from these data, it would appear that at experimental concentrations, curcumin can effectively attenuate the damage in TNBS-induced colitis.

### Histological improvement in response to curcumin

**Fig. 2:**
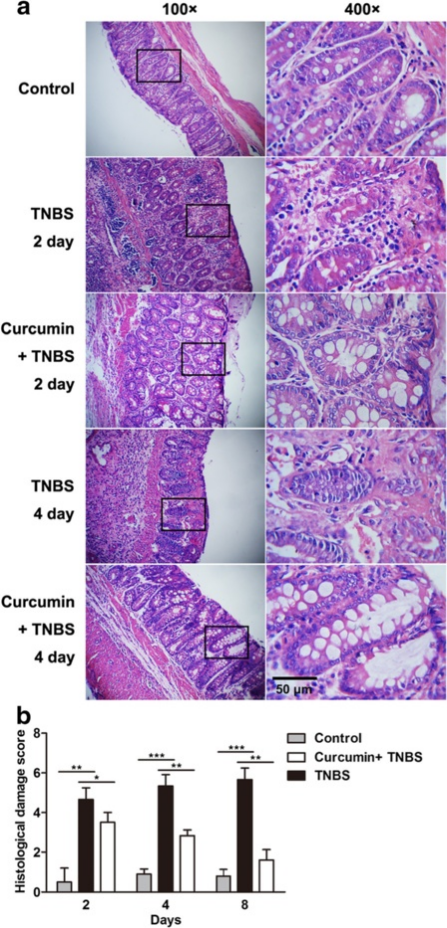
Histological improvement in response to curcumin in TNBS-induced IBD. **a** After TNBS treatment at indicated time, colons were isolated and harvested. With H&E staining, the histological changes were observed. The damage including mucosal ulceration, thickened wall, and inflammatory cells infiltration occurred in the TNBS-treated group, however, the damage was reduced with curcumin pretreatment. **b** Histological damage score were evaluated with time course. There were 20 rats were evaluated for each score. *P < 0.05, **P < 0.01, ***P < 0.001

The colons were isolated and sectioned. After fixing, sections were stained with H&E. The representative sections were selected and shown in Fig. 2a, as expected, curcumin pretreatment could alleviate inflammatory cellular infiltrate, mucosal ulceration, as well as wall thickening which were usually observed in TNBS-induced colitis. In addition, the effect of curcumin on histological damage was shown in Fig. 2b, and clearly revealed that colonic inflammation was dramatically reduced with curcumin treatment, but remained severely inflamed in TNBS-induced colitis 2 days postinduction of colitis. After 4 and 8 days induction of colitis, in curcumin + TNBS group, there appeared to be a trend toward totally improvement as control group. Therefore, histological analysis indicates that curcumin plays a critical role in improving histological phenotype in TNBS- induced colitis.

### Reduced pro-inflammatory markers/cytokines and increased anti-inflammatory cytokines with curcumin pretreatment

**Fig. 3:**
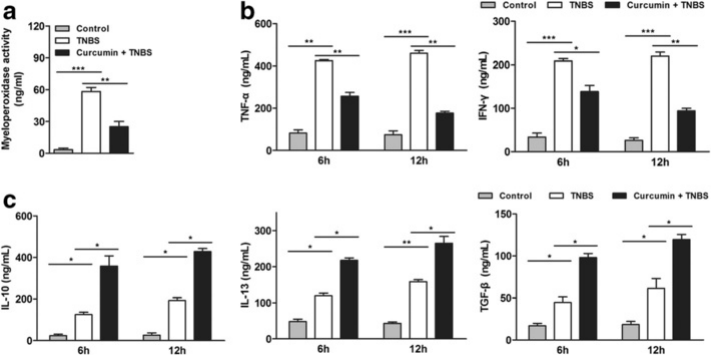
curcumin pretreatment reduced the levels of pro-inflammatory markers/ cytokines but increased the levels of anti-inflammatory cytokines. **a** Curcumin pretreatment inhibited the TNBS-induced increase of MPO activity. **b** The levels of pro-inflammatory cytokines TNF-α and IFN-γ were assessed by ELISA. Curcumin pretreatment inhibited the levels of TNF-α and IFN-γ. **c** The levels of anti-inflammatory cytokines IL-13, TGF-β and IL-10 were assessed by ELISA. Curcumin pretreatment elevated the levels of IL-13, TGF-β and IL-10. *P < 0.05, **P < 0.01, ***P < 0.001

As we known, myeloperoxidase activity is an established marker for inflammatory cell (mainly neutrophils) infiltration and activity in murine models of colitis. TNBS group showed severe neutrophils infiltration at 4 days postinduction of colitis. In contrast, an obviously reduced colonic myeloperoxidase activity with curcumin pretreatment (Fig. 3a), indicating that curcumin inhibits inflammatory response.

Besides, we detected the level of cytokines in peripheral blood which are involved in pro-inflammatory and anti-inflammatory function, including TNF-α, IFN-γ, IL-10, IL-13, TGF-β. TNBS could induce the increase of pro-inflammatory factors TNF-α, IFN-γ, however, there was a significant reduction of TNF-α, IFN-γ in curcumin + TNBS group (Fig. 3b) in a time- dependent manner. It suggests that curcumin has an inhibition effect on inflammation. For the anti-inflammatory cytokines, including IL-10, IL-13, TGF-β, a slight increase was observed in TNBS-induced colitis, which was consistent with previous work [19]. However, in curcumin + TNBS group, the levels of IL-10, IL-13, TGF-β in peripheral blood were significantly elevated (Fig. 3c), implying curcumin is benefit for anti-inflammatory activity. Therefore, these results further confirm the curcumin appears anti-inflammatory effect in TNBS-induced IBD.

### Curcumin declined M1/M2 macrophage ratio in colon

**Fig. 4:**
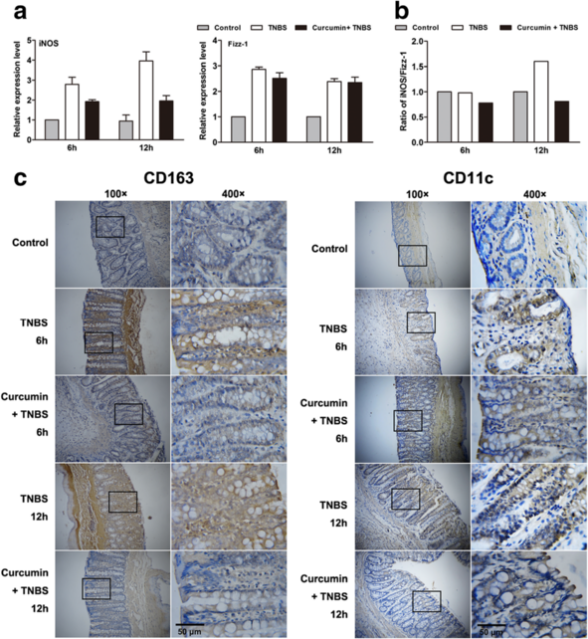
Curcumin pretreatment declined M1/M2 macrophage ratio in colon. **a** The mRNA levels of iNOS and Fizz-1 were detected by qRT-PCR. iNOS and Fizz-1 are the markers of M1 and M2. **b** Curcumin led to the ratio of iNOS/Fizz-1 declined. **c** Representative images of CD11c staining (left sections) and CD163 staining (right sections) in colonic tissue from each group. Curcumin pretreatment decreased the areas of CD11c and CD163 staining

M1, termed as classically activated macrophages, have been associated with inflammatory products such as TNF-α and inducible nitric oxide synthase (iNOS) [20]. By contrast, alternative activated macrophages (M2) inhibit the production of pro-inflammatory cytokines and make contributions to wound healing and tissue repair, which were associated with high arginase 1 (Arg1), Fizz-1 expression [21]. Colon from TNBS group had an extremely high level of iNOS, but with curcumin pretreatment, the increased level was inhibited. In contrast, the level of Fizz-1 in colon in TNBS + curcumin group was higher than in TNBS group (Fig. 4a). Analysis of the ratio of iNOS/Fizz-1 mRNA levels can be as an indicator of the M1/M2 activity balance [22]. As shown in Fig. 4b, curcumin pretreatment skewed this ratio toward Fizz-1 expression, but in TNBS group, cells tended to express more iNOS. Since iNOS and Fizz-1 are the well known markers of M1 and M2, these results suggest curcumin promotes the M2 macrophage polarization.

Similarly, the levels of CD11c and CD163 as an index of M1 and M2 surface marker [23] were also observed by immunostaining, in consistent with the level of iNOS and Fizz-1, there were decreased infiltration of CD11c (M1 macrophage marker) - positive cells, but the similar infiltration of CD163 (M2 macrophage marker) - positive cells were shown in colon in TNBS + curcumin group (Fig. 4c), compared with TNBS group. Take together, it suggests that curcumin declines M1/M2 macrophage ratio in colon, trends toward M2 macrophage polarization, and finally has anti-inflammation effect in colitis.

### The effect of curcumin on anti-inflammation mediated by SOCS-1

**Fig. 5:**
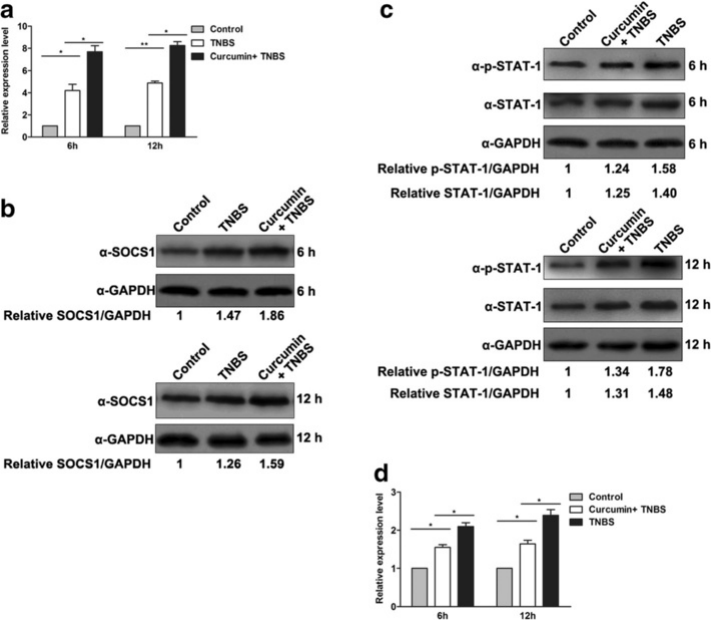
The effect of curcumin on anti-inflammation was mediated by SOCS-1. **a** The mRNA levels of SOCS-1 were detected by qRT-PCR. Curcumin pretreatment increased the transcription of SOCS-1. **b** The protein levels of SOCS-1 were detected by Western Blot at 6 and 12 h postinduction. Curcumin pretreatment increased the protein level of SOCS-1. **c** The total STAT1 proteins and p-STAT1 were detected by Western Blot. The level of p-STAT1 was declined with curcumin treatment. **d** The mRNA levels of STAT1 were detected by qRT-PCR. Curcumin pretreatment suppressed the transcription of STAT1

The suppressor of cytokine signaling-1 (SOCS-1) is a potent negative regulator of various cytokines and it has been implicated in the regulation of immune responses [24]. Previous work has demonstrated that SOCS-1 plays an important role in preventing murine colitis by restricting the cytokine signals [25], however, whether the effect of curcumin on anti-inflammation in TNBS-induced IBD is mediated by SOCS-1 has not been clarified. To this end, we detected the mRNA and protein level of SOCS-1 in colon. An efficiently stimulative effect of curcumin was observed on SOCS-1 in a time-dependent manner (Fig. 5a, b), suggesting SOCS-1 is involved in the improvement of curcumin on TNBS-induced IBD.

SOCS-1 has been identified as a negative feedback regulator of JAK-STAT signaling [26], while IFN-γ induces the phosphorylation of STAT1 so as to activate the signal pathway. We have validated that curcumin can suppress the level of IFN-γ (Fig. 3b), as expected, phosphorylation of STAT1 was indeed decreased with curcumin pretreatment in colon, especially after 12 h TNBS stimulation (Fig. 5c). Furthermore, the mRNA level of STAT1 and total STAT1 proteins were both declined with curcumin treatment in colon (Fig. 5c and d). Taken together, curcumin promote the SOCS-1 expression, the increased SOCS-1 and decreased IFN-γ coordinately impaire the JAK-STAT signaling.

### Curcumin reduces cell apoptosis in colon

**Fig. 6:**
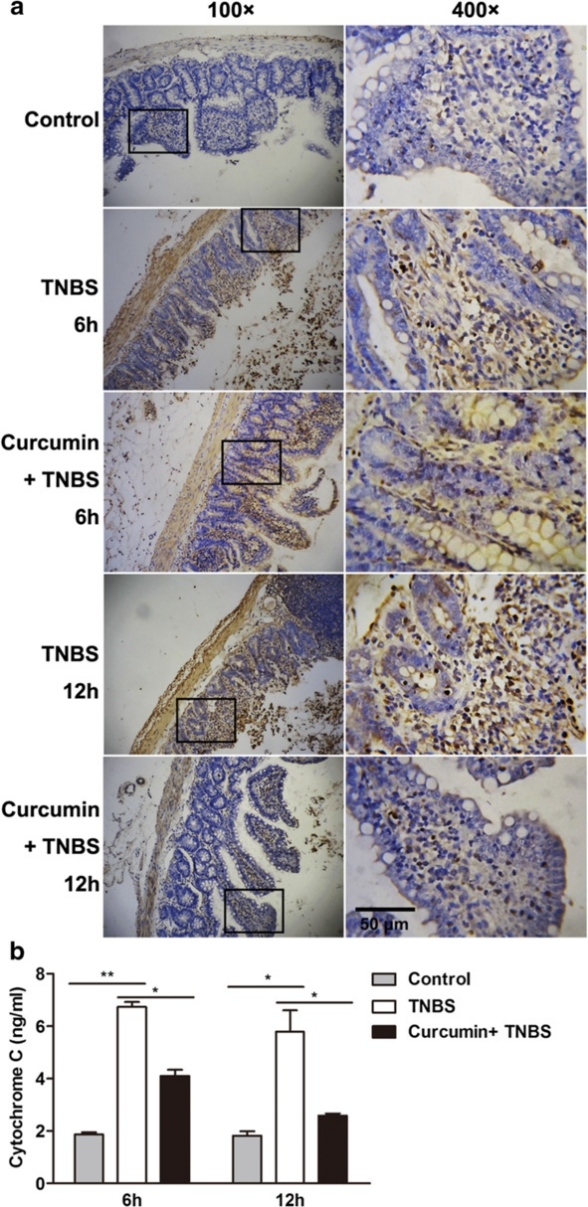
Cell apoptosis reduced with curcumin pretreatment in TNBS-induced colitis. **a** Representative TUNEL stains in colon tissues obtained from rats in each group. Curcumin pretreatment showed fewer areas of TUNEL stains compared with the only TNBS induction. **b** The level of cytochrome C was detected as described in MATERIALS AND METHODS. Cytochrome C declined in curcumin-pretreated rats

Previous study has demonstrated that curcumin can induce a p53-dependent apoptosis in human carcinoma cells [27]. To validate the positive or negtive effect of curcumin on apoptosis in TNBS-induced IBD, TUNEL staining was performed. When frozen sections of colons from rat without TNBS-induced were stained by TUNEL, nearly no signal were detected (Fig. 6a), indicating apoptotic cells was less in normal colon. However, after TNBS treatment, large amounts of TUNEL-positive cells were detected in the crypts and lamina propria of colons. In addition, granulocytes infiltrated into lamina propria (Fig. 6a). Colons from rat pretreatment with curcumin contained abundant TUNEL-stained cells after 6 h induction, but at 12 h postinduction less TUNEL-stained cells were observed, suggesting curcumin can reduce cell apoptosis in TNBS-induced IBD.

In addition, induction of apoptosis by curcumin is through cytochrome C release and activation of caspases in human leukemia HL-60 cells [28]. To clarify this process also occurs in TNBS-induced colitis, the amount of cytochrome C, a mitochondrial apoptogenic protein, in cytosol was detected. Rats treated with TNBS appeared to be cytosolic accumulation of cytochrome C, while the release of cytochrome C was declined in colon in curcumin-pretreated rat (Fig. 6b). These results indicate the antagonistic effect of curcumin on apoptosis in TNBS-induced colitis is through mitochondrial pathway.

## Discussion

In this study, we evaluated the effects of curcumin in a TNBS-induced rats model of IBD. The results illustrated that curcumin could efficiently inhibit pro-inflammatory cytokines secretion and reduce M1/M2 ratio. The anti-inflammatory effect of curcumin effectively inhibited the increase in iNOS level and the extent of infiltrating neutrophils. In addition, we validated that the inhibition of inflammatory activity was mediated by SOCS-1 as well as suppressing the phosphorylation of STAT-1.

The negative regulation of SOCS-1 on JAK2/STAT phosphorylation was mediated a lot of cytokines [9]. Previous study has demonstrated that curcumin can effectively induce the expression of SOCS-1 through inhibiting the activity of class I histone deacetylases in myeloproliferative neoplasms [12]. We also clarified that curcumin can induce SOCS-1 in TNBS-induced intestinal inflammation. SOCS-1 can directly binds JAK2 [10], and finally degrade JAK2 through E3 ubiquition ligase ECS complex. We detected the decreased phosphorylation of STAT-1 as expected in our model. Therefore, we propose that the repressive effect of curcumin on JAK/STAT signaling is mediated by SOCS-1 in TNBS-induced intestinal inflammation. However, other study has shown curcumin suppresses JAK-STAT signaling via activation of SHP-2 in brain microglial cells [11]. Based on these studies, the inhibition of curcumin on JAK-STAT signaling maybe complex and differently regulated in different cells. Frequent apoptosis is usually observed in colitis which is accompanied by loss of epithelial cells. In an experimental study of colitis in rats, compared with the control group, caspase-3 activity in colon was higher in TNBS treatment group [29]. The relationship between curcumin and apoptosis is controversial. It is said that curcumin can inhibit the apoptosis in human and rat T lymphocytes [30], however, others have an opposite result happened in HL60 cell lines [31] and in vivo in colon tumors [32]. The controversy may due to the difference of incubation periods, the curcumin concentrations, and cell lines. In our experiment, with curcumin pretreatment, we observed a significant attenuated apoptosis in colons, this available data indicate that the protective effects of curcumin against TNBS- induced colitis might be, at least in part, mediated by its anti-apoptotic effects. Moreover, we confirmed that curcumin inhibits TNBS-mediated apoptosis in intestinal inflammation by mitochondrial cytochrome c release.

TNF-α is an important cytokine produced mainly by activated monocytes and macrophages. It has been shown that curcumin can attenuate TNF-α-induced oxidative stress, colitis in rats [33]. The administration of TNF-α antibodies can effectively treat experimental rat colitis [34, 35]. Our results also supported the idea that curcumin causes lower serum level of TNF-α, and the reduced colonic tissue oxidative stress. At last, we observed less apoptosis with curcumin treatment.

Melatonin (5-methoxy-N-acetyltryptamine), a derivative of the essential amino acid tryptophan, is also useful in the treatment of IBD patients. This agent has been identified to have anti-inflammatory and anti-apoptotic effects on IBD, which is the same as curcumin. Therefore, we may propose that the substance which has anti-inflammatory and anti-apoptotic effect may be a potential drug to treat IBD.

## Conclusions

Curcumin has an anti-inflammatory effect which is fulfilled by enhancing SOCS-1 expression and inhibiting JAK/STAT pathways. Curcumin also plays an anti-apoptotic role in TNBS-induced intestinal inflammation. Curcumin may supply potential therapeutic implications for human intestinal inflammation.

## Abbreviations

COX: Cyclooxygenase
H&E: Hematoxylin And Eosin
IBD: Inflammatory Bowel Disease
IFNs: Interferons
iNOS: Inducible Nitric Oxide Synthase
JAK: Janus Kinase
MAPK: Mitogen-Activated Protein Kinase
MPNs: Myeloproliferative Neoplasms
qRT-PCR: Quantitative Real-Time Pcr
SOCS: Suppressor Of Cytokine Signaling
STAT: Signal Transducers And Activators Of Transcription
TNBS: 2,4,6-Trinitrobenzene Sulfonic Acid
TNF-α: Tumor Necrosis Factor-Alpha
